# Author Correction: Crossover between the adiabatic and nonadiabatic electron transfer limits in the Landau-Zener model

**DOI:** 10.1038/s41467-021-21530-8

**Published:** 2021-02-15

**Authors:** Guang Yuan Zhu, Yi Qin, Miao Meng, Suman Mallick, Hang Gao, Xiaoli Chen, Tao Cheng, Ying Ning Tan, Xuan Xiao, Mei Juan Han, Mei Fang Sun, Chun Y. Liu

**Affiliations:** Department of Chemistry, Jinan University, 601 Huang–Pu Avenue West, Guangzhou, 510632 China

**Keywords:** Organometallic chemistry, Electron transfer

Correction to: *Nature Communications* 10.1038/s41467-020-20557-7, published online 19 January 2021.

The original version of this Article contained an error in the equation in the first paragraph of the Introduction, which incorrectly read ‘adiabatic : *ν*_el_ >> *ν*_n_; nonadiabatic : *ν*_el_ << *n*_n_’. The correct version states ‘nonadiabatic : *ν*_el_ << *ν*_n_’ in place of ‘nonadiabatic : *ν*_el_ << *n*_n_’.

The original version of this Article also contained an error in the first sentence of the first paragraph of the ‘Synthesis and characterization of the mixed-valence Mo_2_ dimers’ section of the Results, which incorrectly read ‘4,4′-(EE′C(C_6_H_4_C)_3_EE′)^2-^’. The correct version states ‘4,4′-(EE′C(C_6_H_4_)_3_C EE′)^2−^’ in place of ‘4,4′-(EE′C(C_6_H_4_C)_3_EE′)^2-^’.

The original version of this Article also contained an error in the fifth sentence of the first paragraph of the ‘Optical behaviors of the mixed-valence complexes’ section of the Results, which incorrectly read ‘[OO–(ph)_3_–OO]’. The correct version states ‘[OO–(ph)_3_–OO]^+^’ in place of ‘[OO–(ph)_3_–OO]’.

The original version of this Article also contained an error in the legend title of Fig. 3, which incorrectly read ‘Schematic description of donor–acceptor electron in the phenylene bridged Mo_2_ dimer’. The correct version adds ‘transfer’ after ‘donor–acceptor electron’.

The original version of this Article also contained an error in the first sentence of the third paragraph of the Discussion, which incorrectly read ‘Δ*G* ≫ 2*H*_ab_’. The correct version states ‘ΔG* ≫ 2*H*_ab_’ in place of ‘Δ*G* ≫ 2*H*_ab_’.

These have now been corrected in the PDF and HTML versions of the Article.

