# Supplementary material for: Crossover between the adiabatic and nonadiabatic electron transfer limits in the Landau-Zener model
Source: Nat Commun. 2021 Jan 19;12:456. doi: 10.1038/s41467-020-20557-7 (PMC7815917; doi:10.1038/s41467-020-20557-7)
Supplement: Supplementary file 1 — Supporting Information [file 41467_2020_20557_MOESM1_ESM.pdf]

Supplementary Information for：

Crossover between the Adiabatic and Nonadiabatic Electron Transfer Limits in the Landau-Zener model

Guang Yuan Zhu, † Yi Qin, † Miao Meng, † Suman Mallick, Hang Gao,
Xiaoli Chen, Tao Cheng, Ying Ning Tan, Xuan Xiao, Mei Juan Han, Mei Fang Sun and Chun Y. Liu*

Correspondence to: E-mail: tcyliu@jun.edu.cn

Table of Contents

1. Supplementary materials and methods

2. Electrochemical characterization

3. X-ray structural determination

4. Electron paramagnetic resonant (EPR) characterization

5. Electronic and vibronic spectroscopies

6. Optical analysis of the IVCT parameters

7. Supplementary references

1. Materials and methods

Synthesis of dimolybdenum starting materials. All manipulations were performed in a nitrogen-filled glove box or by using standard Schlenk-line techniques. All solvents were purified using a Vacuum Atmosphere (VAC) solvent purification system or freshly distilled over appropriate drying agents under nitrogen. HDAniF[1] and $Mo_2(DAniF)_3(O_2CCH_3)$[2] were synthesized according to the literature methods.

Preparation of 4,4'-terphenyldithiodicarboxylic acid. A solution of 4,4'-terphenyldicarboxylic acid (0.95, 3 mmol) in thionyl chloride (15 mL) with addition of DMF (1 mL) was refluxed for 12 h. Then the solvents were removed under reduced pressure and the residue was mixed with thioacetamide (0.6 g, 8 mmol) in THF (20 mL). The mixture was allowed to stir for 5 h at room temperature. Dilute NaOH solution (10%, 30 mL) was added slowly and further stirred for 30 min. After removal of the solvents, the residue was treated with dilute hydrochloric acid (1 M, 30 mL). The mixture was extracted with diethyl ether (3 × 30 mL) and the combined organic layers were dried over $MgSO_4$. Removal of the solvents under reduced pressure gave the product 4,4'-terphenyldithiodicarboxylic acid as a yellow solid. Yield of **4,4'-terphenyldithiodicarboxylic acid**: 0.85 g, 81%.

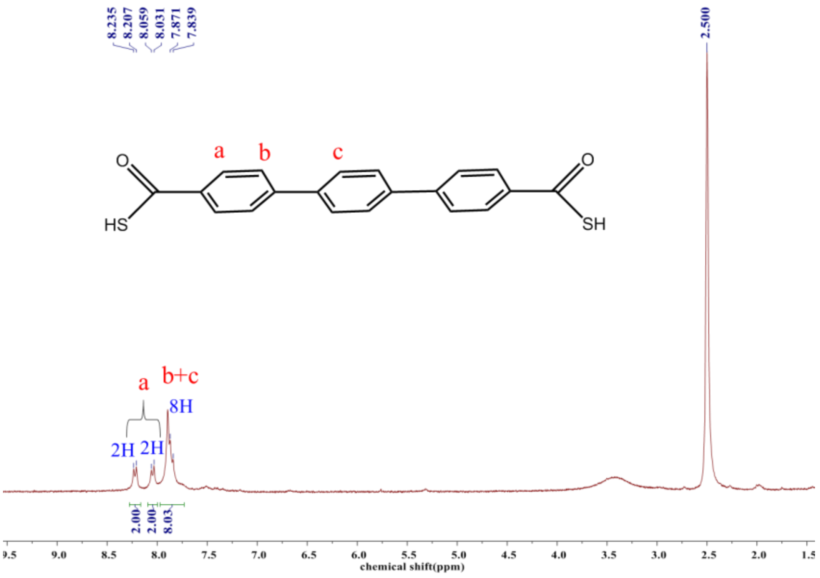

Supplementary Figure 1| ^{1}H NMR spectrum of **4,4'-terphenyldithiodicarboxylic acid** in DMSO-d_6. ^{1}H NMR δ (ppm in DMSO-d_6): 8.23 (d, 2H, aromatic C–H), 8.06 (d, 2H, aromatic C–H), 7.87 (d, 4H, aromatic C–H), 7.84 (d, 4H, aromatic C–H).

Preparation of 4,4'-terphenyltetrathiodicarboxylic acid. Sulfur (0.80 g, 25 mmol) and sodium methoxide (1.21 g, 22.4 mmol) were mixed in 30 mL of methanol. The mixture was then refluxed for 3 h. 4,4'-bis(bromomethyl)terphenyl (2.33 g, 5.6 mmol) was added in portions over a period of 1 h. The reaction mixture was allowed to reflux for additional 12 h, generating a red solution with some solids suspended. The

solution was cooled to room temperature and filtered through a crucible funnel. Then dilute hydrochloric acid was added dropwise to the filtrate and the resultant 4,4'-terphenyltetrathiodicarboxylic acid was extracted using 30 mL of dichloromethane. Removal of the solvents under reduced pressure gave purple colored product which was collected and dried under vacuum.

Yield of **4,4'-terphenyltetrathiodicarboxylic acid**: 1.23 g, 57%.

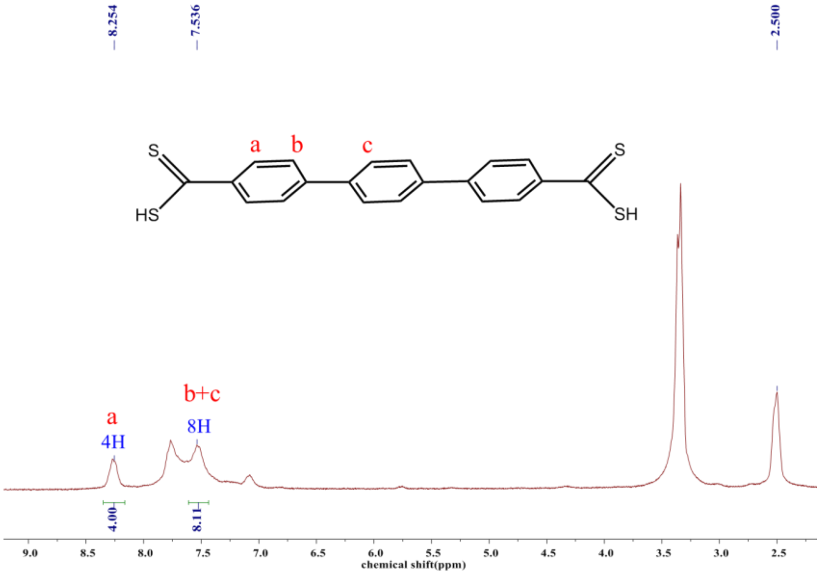

Supplementary Figure 2| ^{1}H NMR spectrum of **4,4'-terphenyltetrathiodicarboxylic acid** in DMSO-d_6. ^{1}H NMR δ (ppm in DMSO-d_6): 8.27 (d, 4H, aromatic C–*H*), 7.52 (d, 8H, aromatic C–*H*).

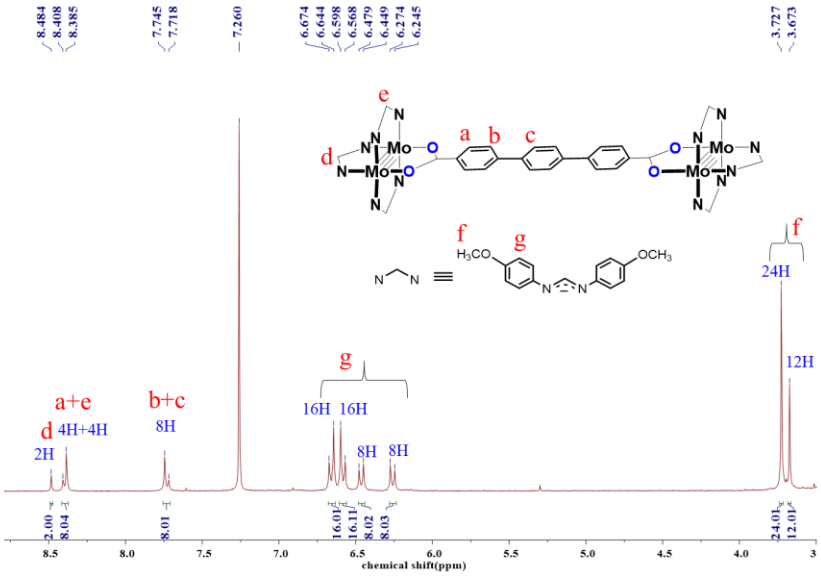

Supplementary Figure 3| ^{1}H NMR spectrum of [**OO–(ph)$_3$–OO**] in $CDCl_3$. ^{1}H NMR δ (ppm in $CDCl_3$): 8.49 (s, 2H,–NC*H*N–), 8.41 (d, 4H, terphenyl C–*H*), 8.39 (s, 4H, –NC*H*N–), 7.75 (d, 4H, terphenyl C–*H*), 7.72 (d, 4H, terphenyl C–*H*), 6.67 (d, 16H, aromatic C–*H*), 6.60 (d, 16H, aromatic C–*H*), 6.50 (d, 8H, aromatic C–*H*), 6.27 (d, 8H, aromatic C–*H*), 3.73 (s, 24H, –OCH_3), 3.67 (s, 12H, –OCH_3).

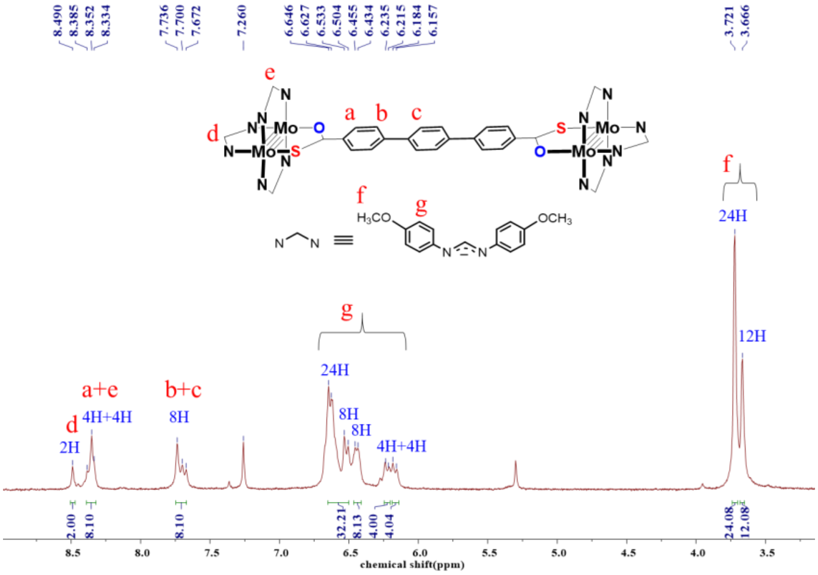

Supplementary Figure 4| ^{1}H NMR spectrum of **[OS–(ph)$_3$–OS]** in $CDCl_3$. ^{1}H NMR δ (ppm in $CDCl_3$): 8.49 (s, 2H, –NC*H*N–), 8.38 (d, 4H, terphenyl C–*H*), 8.33 (s, 4H, –NC*H*N–), 7.74 (d, 4H, terphenyl C–*H*), 7.70 (d, 4H, terphenyl C–*H*), 6.64 (d, 24H, aromatic C–*H*), 6.53 (d, 8H, aromatic C–*H*), 6.45 (d, 8H, aromatic C–*H*), 6.23 (d, 4H, aromatic C–*H*), 6.18 (d, 4H, aromatic C–*H*), 3.72 (s, 24H, –OCH_3), 3.66 (s, 12H, –OCH_3).

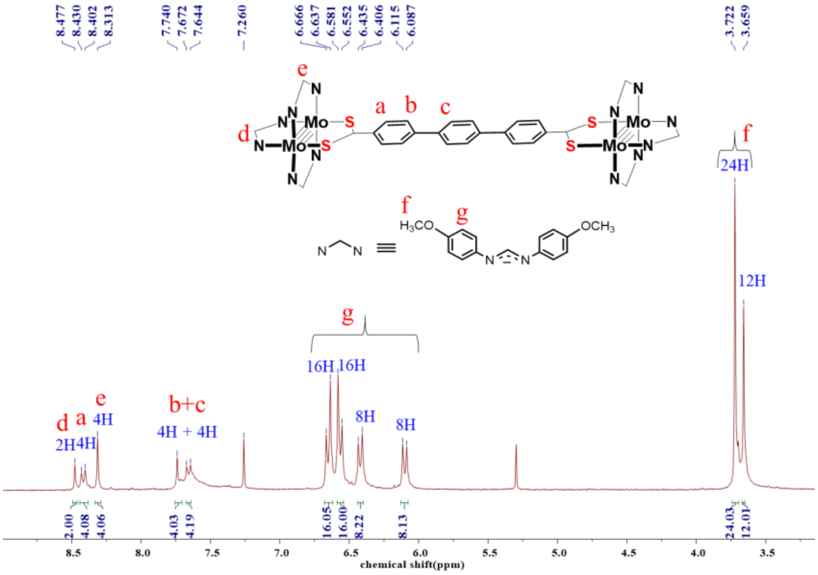

Supplementary Figure 5| ^{1}H NMR spectrum of **[SS–(ph)$_3$–SS]**in $CDCl_3$. ^{1}H NMR δ (ppm in $CDCl_3$): 8.48 (s, 2H, –NC*H*N–), 8.43 (d, 4H, terphenyl C–*H*), 8.31 (s, 4H, –NC*H*N–), 7.74 (d, 4H, terphenyl C–*H*), 7.64 (d, 4H, terphenyl C–*H*), 6.67 (d, 16H, aromatic C–*H*), 6.58 (d, 16H, aromatic C–*H*), 6.44 (d, 8H, aromatic C–*H*), 6.11 (d, 8H, aromatic C–*H*), 3.72 (s, 24H, –OCH_3), 3.66 (s, 12H, –OCH_3).

2. Electrochemical characterization

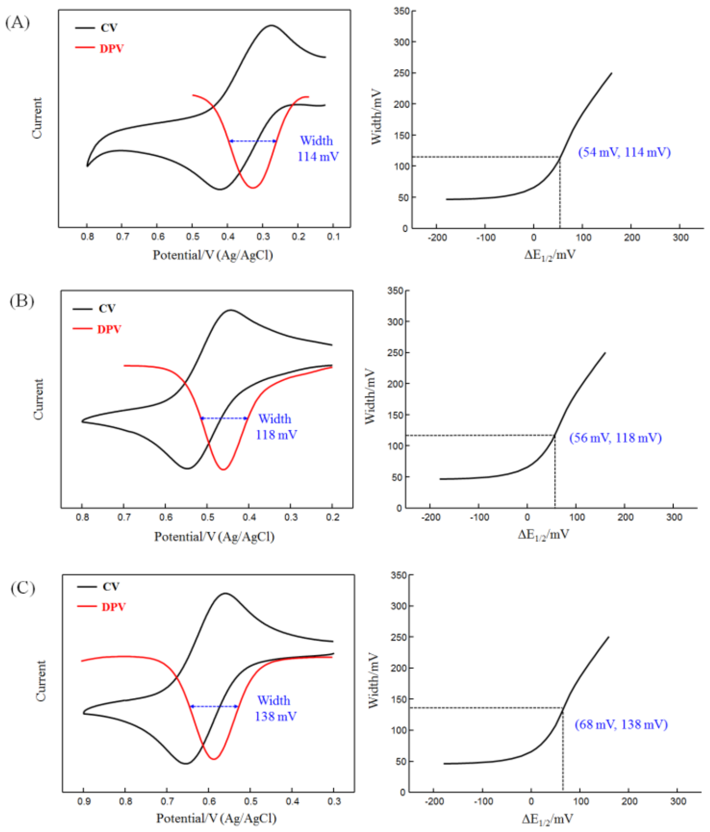

Supplementary Figure 6| Differential pulse voltammograms (DPVs, red) and cyclic voltammograms (CVs, black) for complexes [**OO–(ph)$_3$–OO**] (A), [**OS–(ph)$_3$–OS**] (B), and [**SS–(ph)$_3$–SS**] (C) in 0.1M ^{n}Bu$_4$NPF$_6$/DCM solutions. The $\Delta E_{1/2}$ values are estimated from the working curve (width versus $\Delta E_{1/2}$) based on the Richardson–Taube method.

3. X-ray structural determination

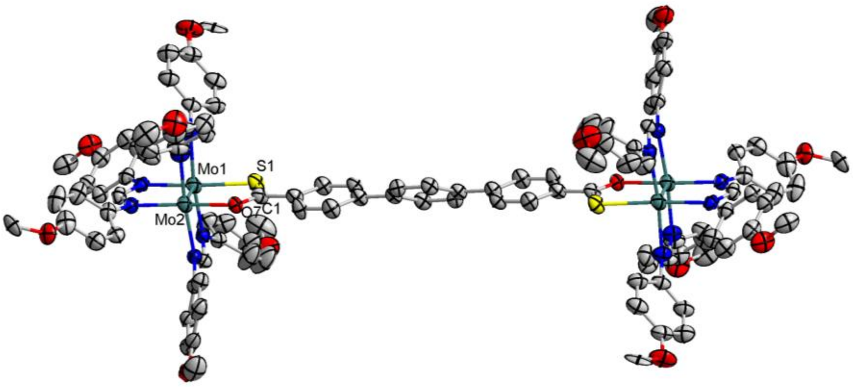

Supplementary Figure 7| X-ray crystal structure of [**OS–(ph)$_3$–OS**] drawn at the 40% ellipsoid probability level. All hydrogen atoms have been omitted for clarity.

Supplementary Table 1| Crystallographic Data for [**OS–(ph)$_3$–OS**].

	[**OS−(ph)$_3$−OS**]·2CH_2Cl_2
formula	$C_{222}H_{208}Cl_4Mo_8N_{24}O_{28}S_4$
FW	2421.67
space group	$P2_1/n$
a(Å)	15.7979(4)
b(Å)	20.0012(5)
c(Å)	16.7675(3)
α(deg)	90
β(deg)	95.4330(18)
γ(deg)	90
V(Å^3)	5274.4(2)
Z	1
T (K)	150
d_{calcd}(g/cm^3)	1.525
μ(mm^{-1})	5.679
R_1^a	0.0890
wR_2^b	0.2631

$^aR_1 = \Sigma||F_o| - |F_c||/\Sigma|F_o|$.
$^bwR_2 = [\Sigma[w(F_o^2 - F_c^2)^2]/\Sigma[w(F_o^2)^2]]^{1/2}$

4. Electron paramagnetic resonant (EPR) characterization

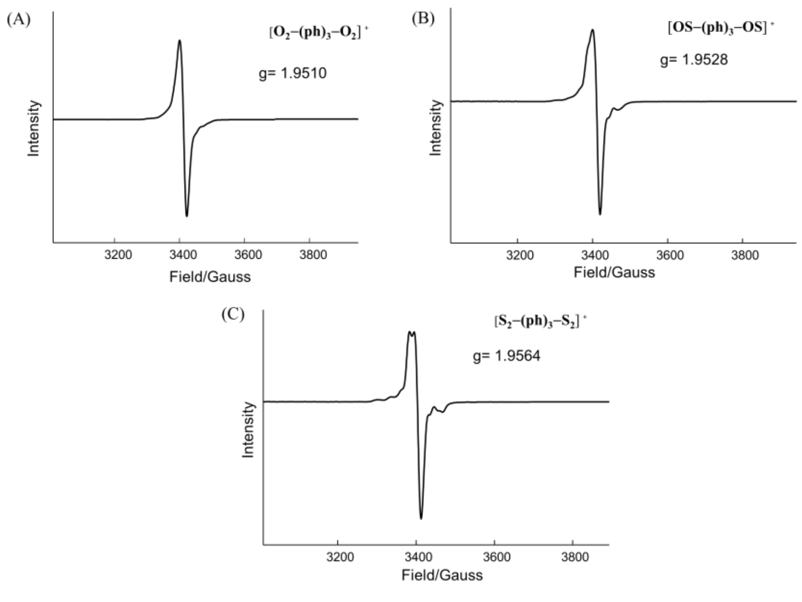

Supplementary Figure 8| X-band EPR spectra for [**OO–(ph)$_3$–OO**]$^+$ (A), [**OS–(ph)$_3$–OS**]$^+$ (B), and [**SS–(ph)$_3$–SS**]$^+$ (C), measured *in situ* in CH_2Cl_2 solutions at 110 K. In the spectra, some hyperfine structures obtained due to less abundant ^{95,97}Mo (I = 5/2) isotopes.

5. Electronic and vibronic spectroscopies

For the neutral bridged dimers, the observed electronic transitions can be unambiguously assigned to the metal-based δ→δ*vertical transition occurring in the Mo_2 center and the metal (δ) to bridging ligand (π*) charge transfer (MLCT). Upon one electron oxidation, the MV species exhibits usually δ→δ* and MLCT bands with the same maximum absorptions and an increased intensity for the δ→δ* transition but reduced intensity for the MLCT band.[3,4] For strongly coupled [**OS-ph-OS**]$^+$ and [**SS-ph-SS**]$^+$, bridging ligand (π) to metal (δ) occur on the cationic Mo_2 center (acceptor), which is absent for the neutral complexes.[3] The UV-Visible spectral data for the neutral [**EE′-(ph)$_n$-EE′**] complexes, listed in Supplementary Table 2, can be used to calculate the electronic constants H_{CNS} according to CNS model.[5] The striking optical feature for the MV complexes [**EE′-(ph)$_n$-EE′**]$^+$ is a low-energy, broad IVCT band with the vibronic transition energy (E_{IT}) spanning from near-IR to med-IR regions. The transition energy, absorption intensity and band shape vary depending on the strength of electronic coupling between the bridged Mo_2 centers. These optical parameters are utilized to derive the electronic coupling constants H_{ab} from the Mulliken-Hush formalism (see Table 1 in the test). (see Supplementary Figure 10-19)

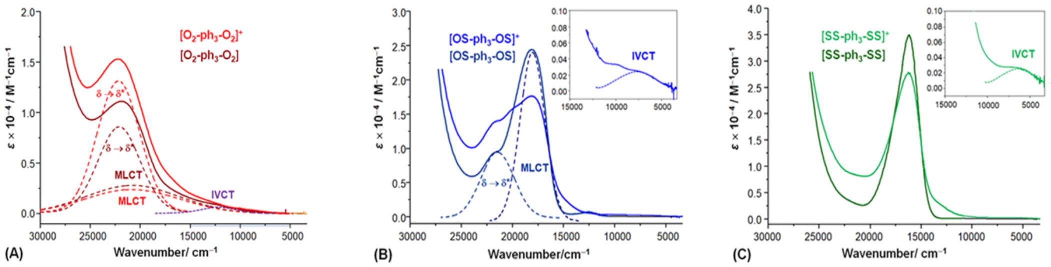

Supplementary Figure 9| UV-Vis-near IR spectra of the triphenylene bridged Mo_2 dimers in neutral (lighter) and mixed-valence (darker) states. (A) [**OO-(ph)$_3$-OO**], (B) [**OS-(ph)$_3$-OS**] and (C) [**SS-(ph)$_3$-SS**]. The insets in (B) and (C) show the IVCT bands for MV complexes [**OS-(ph)$_3$-OS**]$^+$ and [**SS-(ph)$_3$-SS**]$^+$, respectively. For [**OO-(ph)$_3$-OO**] and [**OS-(ph)$_3$-OS**], the spectra were deconvoluted using Gaussian-shaped band profiles to resolve the coalesced δ→δ* and MLCT and the IVCT bands (dashed lines). Optical analysis for deriving the H_{ab} or H_{CNS} are given below (Supplementary Figure 16 and 17, respectively).

6. Optical analysis of the IVCT parameters

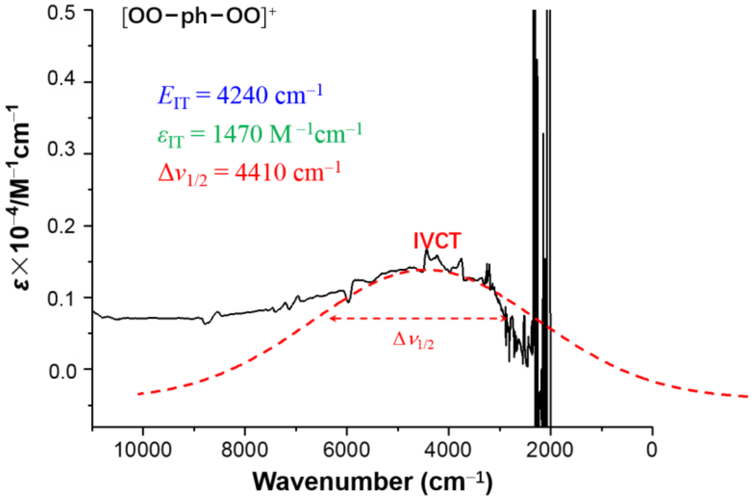

Supplementary Figure 10| IR spectrum of **[OO–ph–OO]**$^{+}$ showing the slightly asymmetrical IVCT absorption band. The IVCT band parameters (E_{IT}, ε_{IT} and $\Delta\nu_{1/2}$) were extracted from the Gaussian-shape simulated band profile (red dashed line) of the vibronic absorption (black solid line). The H_{ab} was calculated from the Mulliken-Hush expression.

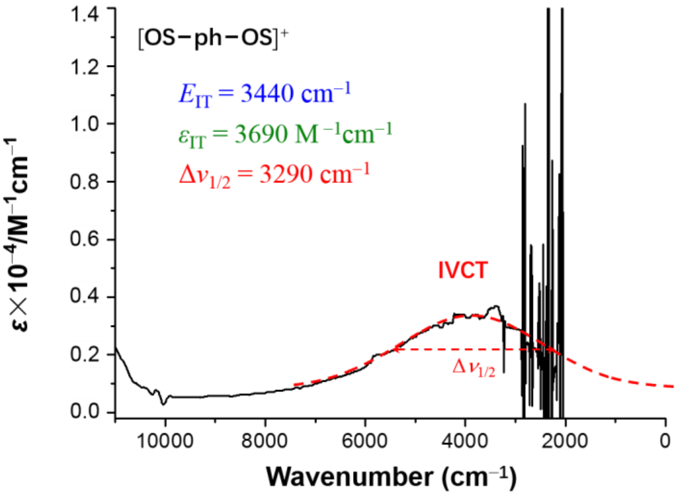

Supplementary Figure 11| IR spectrum of **[OS–ph–OS]**$^{+}$ showing the asymmetrical IVCT absorption band. The IVCT band parameters (E_{IT}, ε_{IT} and $\Delta\nu_{1/2}$) were extracted from the vibronic absorption (black solid line). The H_{ab} was calculated from the Mulliken-Hush expression.

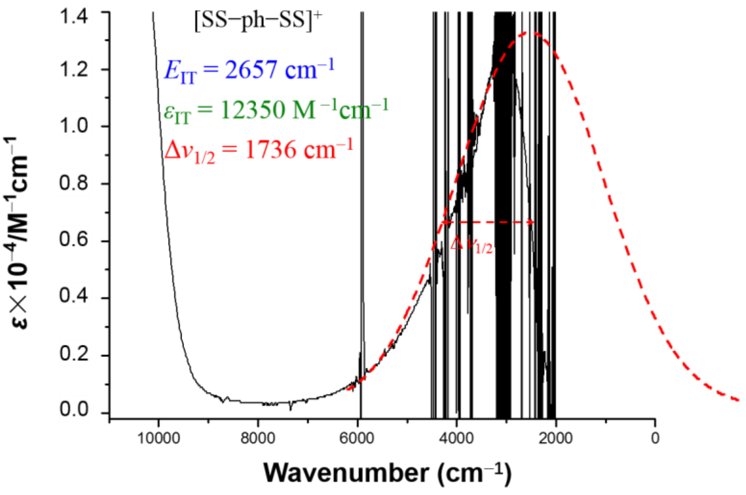

Supplementary Figure 12| IR spectrum of **[SS–ph–SS]**$^+$ showing the half cutting-off IVCT absorption band. The IVCT band energy (E_{IT}) and intensity (ε_{IT}) were measured from the maximum of the Gaussian-shape simulated band profile (red dashed line) and bandwidth $\Delta\nu_{1/2}$ was determined from the vibronic absorption (black solid line). The peak of the IVCT band is cut by the intense vibrational bands. The H_{ab} was calculated from the Mulliken-Hush expression.

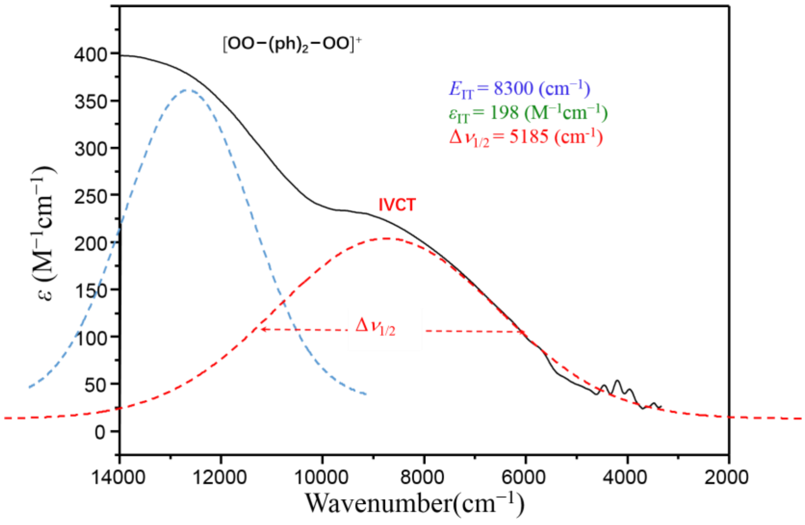

Supplementary Figure 13| Near-IR spectrum of **[OO–(ph)$_2$–OO]**$^+$ showing the IVCT absorption band overlapped with electronic transitions. After deconvolution of the absorbance, the IVCT band parameters (E_{IT}, ε_{IT} and $\Delta\nu_{1/2}$) were extracted from the Gaussian-shape simulated profile (red dashed line) of the vibronic absorption (black solid line). In this work, the measured $\Delta\nu_{1/2}$ of the IVCT band is corrected by deconvolution of the two overlapped bands. The published $\Delta\nu_{1/2}$ value (8300 cm^{-1}) was derived from the spectrum without deconvolution.[4] The H_{ab} in the figure was calculated from the Mulliken-Hush expression using the updated data.

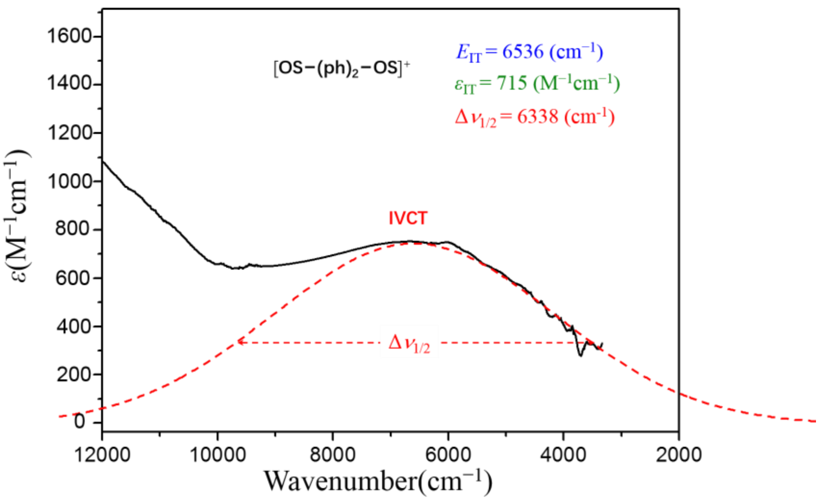

Supplementary Figure 14| Near-IR spectrum of **[OS–(ph)2–OS]**$^+$ showing the IVCT absorption band. The IVCT band parameters (E_{IT}, ε_{IT} and $\Delta v_{1/2}$) were extracted from the Gaussian-shape simulated profile (red dashed line) of the vibronic absorption (black solid line). The H_{ab} was calculated from the Mulliken-Hush expression.

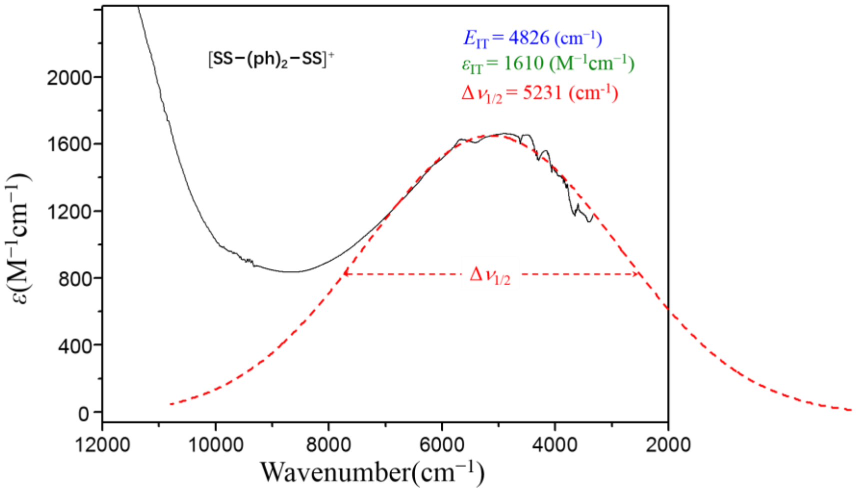

Supplementary Figure 15| Near-IR spectrum of **[SS–(ph)2–SS]**$^+$ showing the IVCT absorption band. The IVCT band parameters (E_{IT}, ε_{IT} and $\Delta v_{1/2}$) are extracted from the Gaussian-shape simulated profile (red dashed line) of the vibronic absorption (black solid line). The H_{ab} was calculated from the Mulliken-Hush expression.

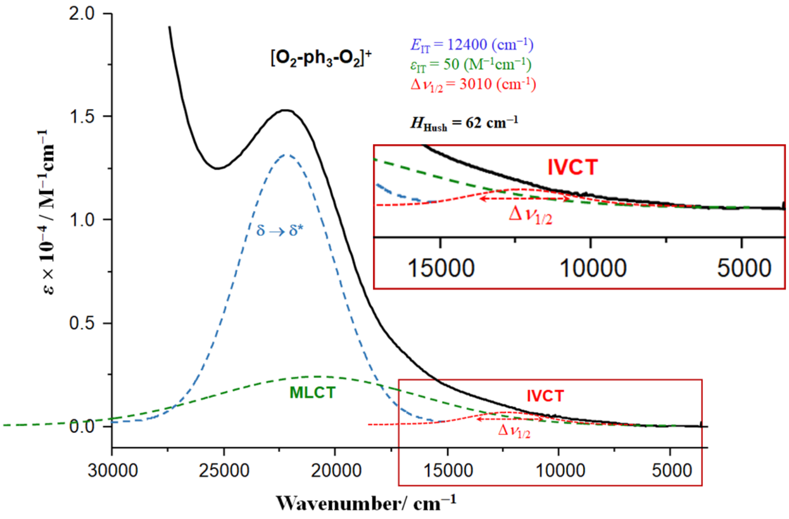

Supplementary Figure 16| Near-IR spectrum of **[OO–(ph)$_3$–OO]**$^+$ showing the IVCT absorption band overlapped with the electronic transitions. The spectrum was deconvoluted into three bands, the δ→δ* transition, the MLCT absorption and vibronic IVCT band based on the known δ→δ* energy (450 nm) and intensity, as shown in Figure. S18 for [**OS-(ph)$_3$-OS**]$^+$. Then, the IVCT band parameters (E_{IT}, ε_{IT} and $\Delta\nu_{1/2}$) are extracted from the by Gaussian-shape simulated profile (red dashed line) of the vibronic absorption (black solid line). Because the IVCT in the spectrum is extremely weak, the E_{IT} of 12400 cm^{-1} was confirmed by extrapolation from those for the ph and ph$_2$ analogues (Supplementary Figure 20). Knowing ε_{IT} = 198 M^{-1}cm^{-1} [**OO-(ph)$_2$-OO**]$^+$ and the great decrease of the IVCT band intensity with extending the bridge to ph$_3$ from ph$_2$ from the other analogues (Table 1 in the test), the measured molar coefficient of 50 M^{-1}cm^{-1} is reasonable, in spite of the uncertainty due to the low intensity and band overlapping. The H_{ab} was calculated from the Mulliken-Hush expression.

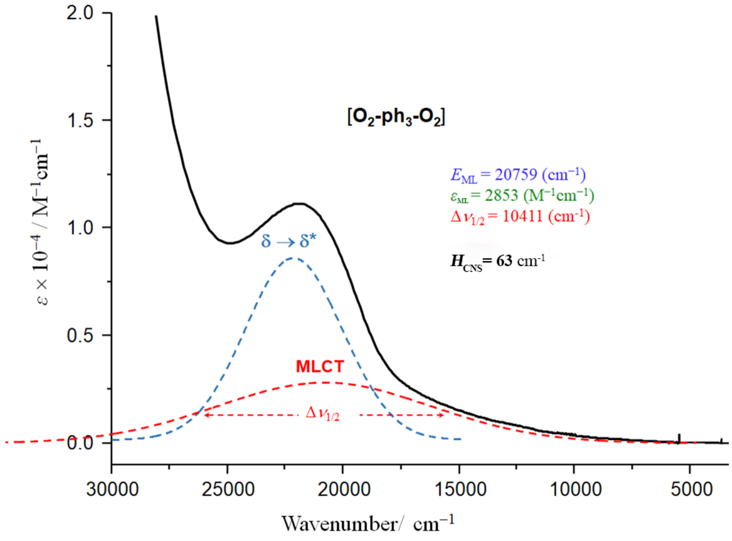

Supplementary Figure 17| UV-Visible spectrum of the neutral **[OO–(ph)3–OO]** showing the overlap of the MLCT band with the δ→δ* transition. After deconvolution of the spectrum, MLCT band parameters (E_{ML}, ε_{ML} and $\Delta v_{1/2}$) are extracted from the Gaussian-shape simulated band profile (red dotted line). With these spectral data, the electronic coupling matrix element, denoted as H_{CNS}, was calculated from the CNS formalism, using equations Supplementary eq 1-3.[5] The H_{CNS} (63 cm^{-1}) is essentially equal to H_{ab} (62 cm^{-1}) derived the Mulliken−Hush expression.

$$H_{MM'} = \frac{H_{ML}H_{M'L}}{2\Delta E_{ML}} + \frac{H_{LM}H_{LM'}}{2\Delta E_{LM}}$$ Supplementary eq 1

$$\frac{1}{\Delta E_{ML}} = 0.5 \times \left(\frac{1}{E_{ML} - E_{\mathrm{IT}}} + \frac{1}{E_{ML}} \right)$$ Supplementary eq 2

$$\frac{1}{\Delta E_{LM}} = 0.5 \times \left(\frac{1}{E_{LM} - E_{\mathrm{IT}}} + \frac{1}{E_{LM}} \right)$$ Supplementary eq 3

Here, H_{ML} and $H_{M'L}$ and H_{LM} and $H_{LM'}$ are the metal to ligand and ligand to metal coupling elements, respectively, for the two sites M−BL and BL−M′ at the geometries associated with the corresponding metal oxidation level.[5] These parameters are calculated from the Mulliken-Hush expressions (eq 2 in the text) using the spectral data from the neutral bridged dimers. ΔE_{ML} and ΔE_{LM} are the effective charge transfer energy gaps. For system **[OO–(ph)3–OO]$^+$** in the absence of LMCT absorption, $H_{LM} = H_{LM'} = 0$ and $\Delta E_{LM} = 0$ are assumed; it is calculated that H_{ML} (= $H_{M'L}$) = 1618 cm^{-1}, ΔE_{ML} = 20759 cm^{-1} and H_{CNS} = 63 cm^{-1}.

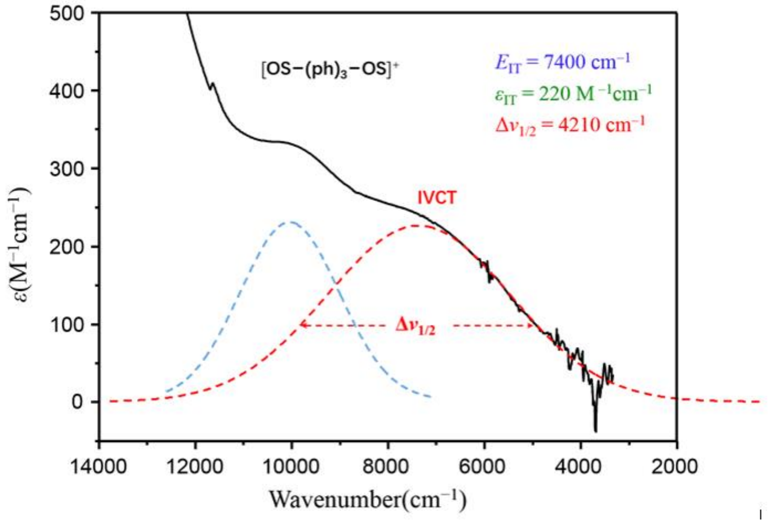

Supplementary Figure 18| Visible-Near-IR spectrum of **[OS–(ph)3–OS]$^+$** showing the IVCT band overlapped with an unidentified absorbance. After deconvolution of the spectrum, the IVCT band parameters (E_{IT}, ε_{IT} and $\Delta v_{1/2}$) were extracted from the

Gaussian-shape simulated band profile (red dashed line) of the vibronic absorption (black solid line). The H_{ab} was calculated from the Mulliken-Hush expression.

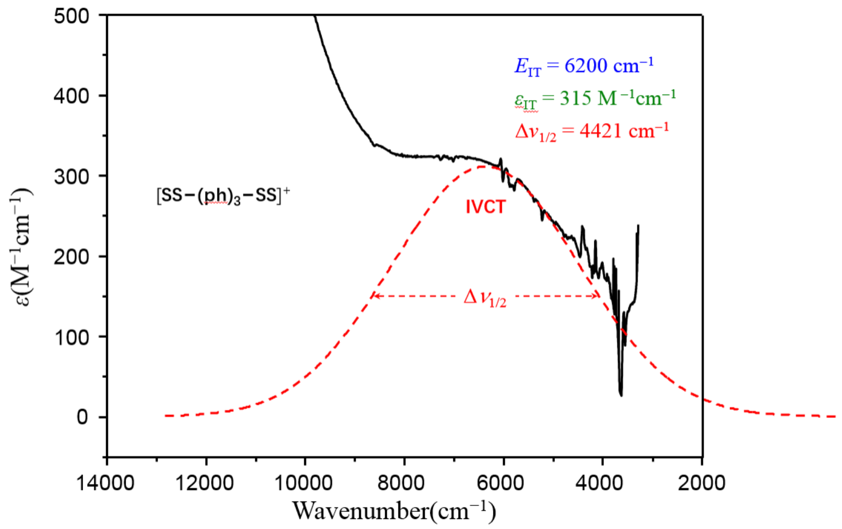

Supplementary Figure 19| Visible-Near-IR spectrum of **[SS–(ph)$_3$–SS]$^+$** showing the IVCT absorption band. The IVCT band parameters (E_{IT}, ε_{IT} and $\Delta\nu_{1/2}$) were extracted from the by Gaussian-shape simulated band profile (red dashed line) of the vibronic absorption (black solid line). The H_{ab} was calculated from the Mulliken-Hush expression.

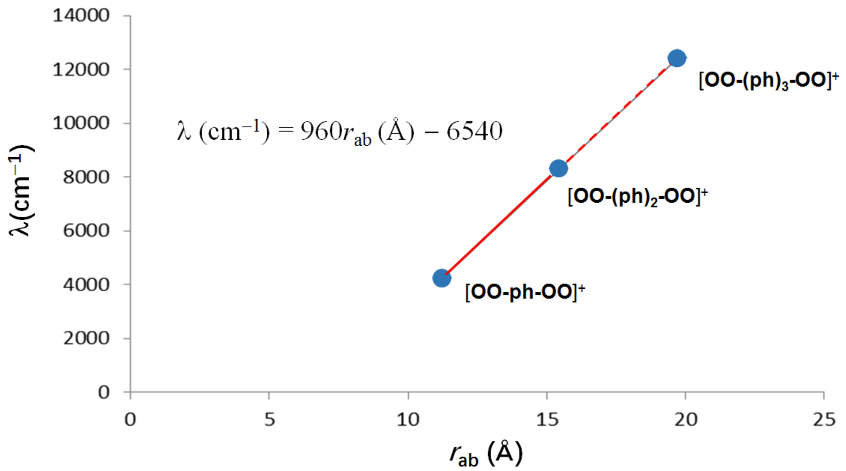

Supplementary Figure 20| Distance-dependence plot of λ versus $r_{c\text{-}c}$ (Å). In symmetrical mixed-valence [Mo_2]-bridge-[Mo_2]$^+$, the IVCT energy (E_{IT} = λ,) is linearly related to the metal-metal separation (r_{ab}). For example, for **[SS-(ph)$_n$-SS]**$^+$ (n = 1-3), λ (cm^{-1}) = 418r_{ab} (Å) – 2154 (R^2 = 0.9812). For **[OO-(ph)$_n$-SS]**$^+$, λ (cm^{-1}) = 960r_{ab} (Å) – 6540, based on the data from the ph and ph$_2$ analogues, from which λ = 12400 cm^{-1} is extrapolated with r_{ab} = 19.74 Å. This value corresponds to the maximum absorption of the IVCT band in the spectrum (Supplementary Figure 16).

Supplementary Table 2| Spectroscopic data of [**EE′−(ph)$_n$− EE′**] and [**EE′−(ph)$_n$−EE′**]$^+$.

complex	n	$r_{e\text{-}e}$ (Å)	[Mo_2]–ph$_n$–[Mo_2]		{[Mo_2]–ph$_n$–[Mo_2]}$^+$		ref
			E_{ML} (nm/cm^{-1})	ε_{ML} ($M^{-1}cm^{-1}$)	E_{ML} (nm/cm^{-1})	ε_{ML} ($M^{-1}cm^{-1}$)	
[**OO-(ph)$_n$-OO**]	1	5.8	485/20619	15230	478/20920	16947	3
	2	10	476/21012	9272	466/21472	17077	4
	3	14.3	446/20759	2853	449/22271	2500	
[**OS-(ph)$_n$-OS**]	1	5.8	623/16051	25870	651/15360	17729	3
	2	10	577/17319	21840	608/16435	13178	4
	3	14.3	550/18182	24517	551/18148	17679	
[**SS–(ph)$_n$–SS**]	1	5.8	722/13850	39960	721/13869	27482	3
	2	10	639/15647	37350	646/15480	23921	4
	3	14.3	611/16367	34807	615/16260	27674	

$^a r_{e\text{-}e}$ is the separation between the two bridged [Mo_2] units, namely, edge to edge distance, which is used for calculation of H_{ab} from the Mulliken-Hush expression.

Supplementary references:

1. C. Lin, J. D. Protasiewicz, E. T. Smith, T. Ren, *Inorg. Chem.* **35**, 6422−6428 (1996).
2. F. A. Cotton, C. Y. Liu, C. A. Murillo, D. Villagrán, X. Wang, *J. Am. Chem. Soc.* **125**, 13564−13575 (2003).
3. C. Y. Liu, X. Xiao, M. Meng, Y. Zhang, M. J. Han, *J. Phys. Chem. C* **117**, 19859−19865 (2013).
4. X. Xiao, M. Meng, H. Lei, C. Y. Liu, *J. Phys. Chem. C* **118**, 8308−8315 (2014).
5. C. Creutz, M. D. Newton, N. Sutin, *J. Photochem. Photobiol. A: Chem.* **82**, 47−59 (1994).
